# Study on deformation and failure characteristics of oblique-cut locked rock slope under rainfall conditions

**DOI:** 10.1038/s41598-024-64329-5

**Published:** 2024-06-19

**Authors:** Hu Wang, Guangxiang Yuan, Zhiquan Huang, Jun Dong, Yi Wei

**Affiliations:** 1 North China University of Water Resources and Electric Power, Henan, 450046 Zhengzhou China; 2 Luoyang Institute of Science and Technology, Henan, 571023 Luoyang China

**Keywords:** Rainfall conditions, Inclined rock slope, Locking section, Failure mode, Numerical simulation, Natural hazards, Geology, Geophysics

## Abstract

Based on the disaster-pregnant environment and development characteristics of landslide disasters in the western region of Henan Province, a generalized model was established by taking the “oblique-cut” locking rock slope in the layered rock slope as the research object. The numerical simulation method was used to analyze the deformation and failure mechanism and stability influence law of the oblique-cut locking rock slope in western Henan under rainfall conditions. The results show that the inclination angle of the weak interlayer directly affects the deformation and failure characteristics of the slope rock mass. With the increase of the geometric parameters of the slope and the inclination angle of the weak interlayer, the failure mechanism is manifested as the slip shear failure along the level at the foot of the slope → the slip shear failure along the level at the foot of the slope (the sliding surface moves upward) → the shear failure in the middle of the slope surface → the slip shear failure along the level at the foot of the slope (the sliding surface moves downward) → the uplift shear failure at the lower edge of the rock layer. When the dip angle of the weak interlayer is constant, the slope stability decreases gradually with the increase in slope gradient and slope height, and the geometric factors of the slope control the overall change trend of the slope stability coefficient. When the slope is greater than 55° and the slope height is greater than 55 m, the shear stress of the slope locking section exceeds its shear strength, and the probability of landslide instability is greatly increased.

## Introduction

Oblique-cut rock landslide refers to the landslide phenomenon where the sliding surface cuts through the rock layer under the action of static aging or dynamic vibration deformation, which is composed of rock and distributed in a certain layer^1^. The overall stability of the oblique rock slope is controlled by the weak interlayer and the trailing edge cracks. Rainfall is an important factor leading to slope instability. Rainfall infiltration is conducive to the extension of slope cracks and greatly reduces the shear strength of weak rocks^2^. From the perspective of region and individual, Rahardjo et al.^3,4^ analyzed the influence of early rainfall on landslide distribution and the influence of rainfall intensity on slope infiltration law. Nguyen et al.^5,6^ studied the spatial variability of shear strength parameters of rainfall-induced landslides, highlighting the importance of each parameter to slope probability analysis. Likitlersuang and Ongpaporn et al.^7–10^ took vegetation roots as the research object and explained the influence of vegetation roots on slope stability under rainfall conditions from multiple perspectives, which provided a new idea for the study of slope instability under rainfall conditions. In recent years, domestic and foreign scholars have carried out some research on the instability mechanism of the cutting rock slope. For example, Guzzetti et al.^11^ conducted a field investigation on the oblique-cutting rock landslides in the Tiber River Basin in central Italy and comprehensively analyzed the development of instability characteristics and failure modes of such slopes. Tiranti et al.^12^ analyzed the stability of the nearly horizontal rock slope in Piedmont Mountain and considered that rainfall was the most important factor leading to the landslide of the nearly horizontal rock layer. Shu et al.^13^ studied the layered rock slope near instability and determined the functional relationship between the slope angle, the friction angle of the failure surface, the cohesion, and the height of the sliding body. Lai et al.^14^ studied and analyzed the stage disaster evolution process of the layered rock landslide and established a geological model. By analyzing the change trend of stress, strain, and displacement in the soil, the deformation and failure mechanisms of the landslide were revealed, which played an early warning role.

**Figure 1:**
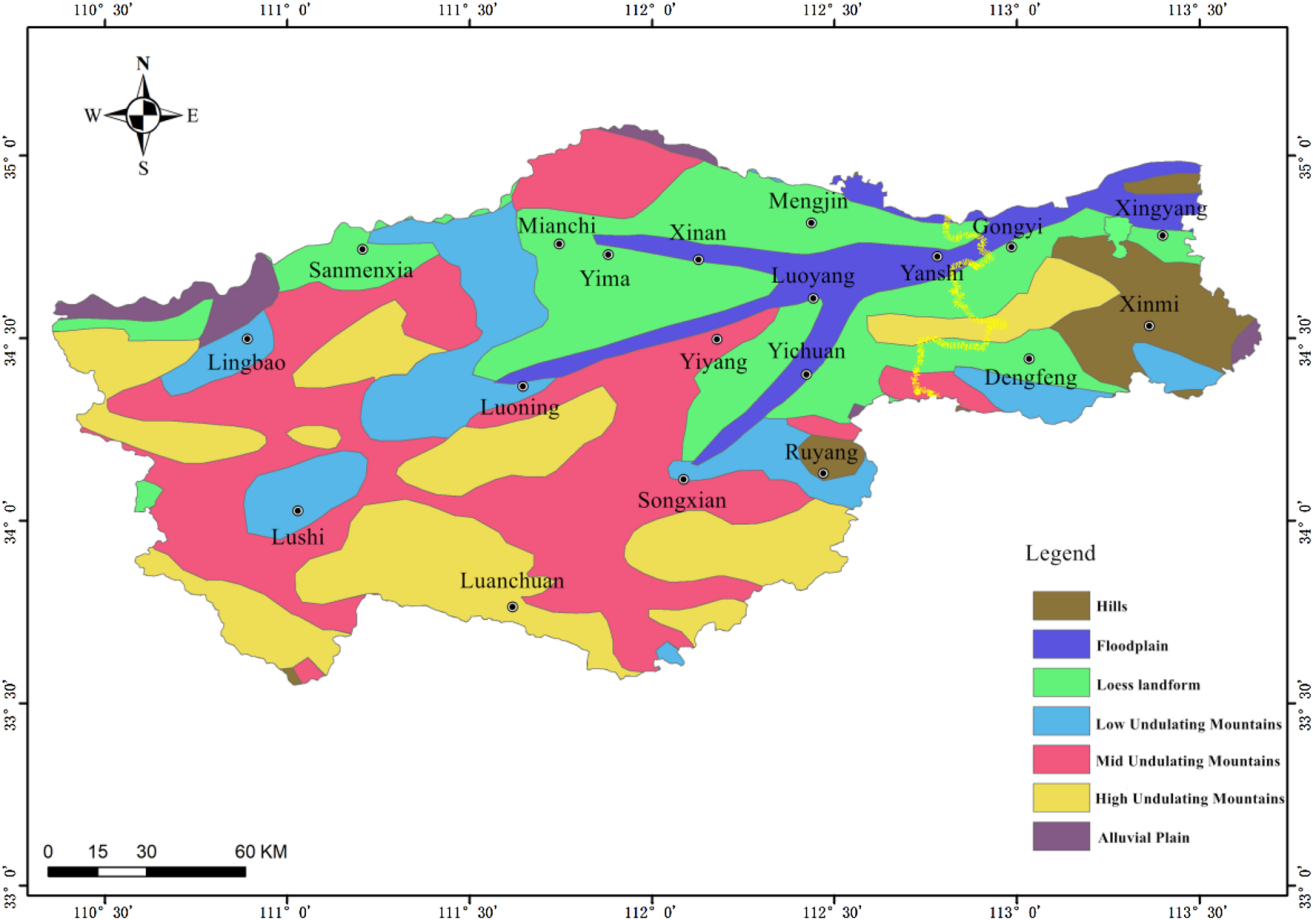
Geomorphic types in western Henan Province (This figure is generated using ArcGIS software-version 10.3.1 https://enterprise.arcgis.com/zh-cn/portal/10.3/use/deploy-app-portal-obsolete.htm).

The western region of Henan Province is located in the transition zone from the second step to the third step of China's terrain. The geological and geomorphological conditions are very complicated (see Fig. 1). The natural geological process and human engineering economic activities are relatively strong. In addition, it is located at the boundary between the northern subtropical and warm temperate climates. Climate, vegetation, hydrology, and soil quality are quite different in the east, west, south, and north regions, which makes the region a landslide-prone area^15^. In July 2021, Zhengzhou City, Henan Province, China, suffered a rare heavy rainstorm in history. The precipitation in most areas was more than 400 mm. The cumulative rainfall in the process was close to or exceeded the annual average rainfall, causing extremely serious urban waterlogging, river floods, flash floods, landslides, and other concurrent disasters, resulting in major casualties and property losses. The disaster level is a particularly major natural disaster^16^. Among them, the four cities in the western mountainous areas of Zhengzhou (Xingyang, Gongyi, Xinmi, and Dengfeng) are the most serious. At 14:00 on July 20, 2021, a landslide occurred in Wangzongdian Village, Cuimiao Town, and three people were buried and killed^17^. The Dongmiaojia landslide at about 2 km downstream of the Xiaolangdi Reservoir dam also caused local deformation and collapse of the slope after the ‘7·20’ heavy rainstorm. The occurrence of these landslides is closely related to the characteristics of the extreme precipitation process, such as long duration, large cumulative rainfall, a wide range of heavy precipitation, and a concentrated and extreme precipitation period^18^.

It has been found that the landslide disasters induced by the above extreme rainfall have the characteristics of sudden releases of high energy. There are local relatively high-strength rock bridges or locking sections in the slope, which play a controlling role in the stability of the landslide. Predecessors call the rock slope with these characteristics a locked rock slope^19^. Some scholars have pointed out that^20^ any part that controls the stability of the slope can be defined as the ‘locked segment’, so the ‘locked segment’ is the key to analyzing the progressive failure of many slopes. Aiming at the locked landslide in the western region of Henan Province, China, Liu Handong et al.^21^ used a self-developed loading device to simulate external loads such as rainfall and earthquakes and carried out the evolution mechanism of the ‘retaining wall’ type locked segment landslide. Li Dongdong et al.^22^ carried out a series of large-scale landslide physical model tests using specific flume test equipment and tested the difference between the failure time and deformation behavior of the three locking section shapes under the same rainfall conditions. However, due to the complexity of meteorological factors and the concealment of the location and type of the locking section in the landslide body, the response and disaster mechanism of geological engineering properties under extreme heavy rainfall are not clear, and it is impossible to judge whether the stable rock slope under normal conditions collapses under heavy rainfall. There are few related studies on the stability, deformation, and failure law of the inclined locking rock slope in the western part of Henan Province under the action of heavy rainfall.

Therefore, this paper takes the oblique-cut locking rock slope in western Henan as the research object and establishes a generalized model of the oblique-cut locking rock slope with reference to the development characteristics of rock landslides in western Henan. The numerical simulation method is used to study the influence of slope geometric characteristics, locking section characteristics, seepage field, and other factors on slope deformation and failure characteristics and instability law under extreme rainfall and then grasp the slope instability initiation mechanism. It provides important support for the identification and disaster prevention of locking rock landslides in western Henan and can play a role in disaster prevention and mitigation, guiding work deployment, and rational land planning, which has important theoretical and practical significance.

## Generalized calculation model and parameter setting

The types of locking sections can be divided into five types: homogeneous rock bridge type, bedding direct shear type, cross-layer oblique cutting type, retaining wall type, and supporting arch type^19^. The stability of the inclined rock slope is controlled by the locking section on the potential sliding surface. The locking section refers to the high-strength part that bears stress concentration, such as the rock bridge and supporting arch^23^. If the locking section of the potential sliding surface of the rock slope runs through, the creep acceleration behavior occurs under the action of rainfall and other factors, and the slope will undergo overall instability and sliding^24^. Siddique et al.^25^ used GIS tools to analyze the spatial variability of RMR, SMR and CSMR technical stability levels, and identified different models that control the failure of jointed rock masses from a kinematic point of view. Kainthola et al.^26^ used the geological strength index (GSI) and Hoek–Brown failure criterion to invert the few input parameters in the finite element analysis, and revealed the deformation mechanics mechanism of the cutting slope. Singh et al.^27,28^ and Verma et al.^29^ used a variety of numerical analysis methods, including finite difference method (FDM), limit equilibrium method (LEM) and finite element method (FEM)—artificial neural network (ANN). The cutting slope in the landslide-prone area of North Akanbangde, India was simulated, and the slope stability was analyzed from multiple perspectives. It provides new inspiration for slope numerical analysis and promotes the development of numerical simulation. Therefore, through numerical simulation, the dynamic evolution characteristics of landslide can be reproduced to a certain extent in a short time, which provides a very good idea for analyzing the dynamic evolution law of landslide. After a reasonable generalization of the oblique-cut locked rock slope, the corresponding geomechanical model is constructed. The dynamic evolution characteristics of the landslide are studied by changing the geometric parameters of the slope and applying rainfall conditions, and then the stability and deformation of the locked rock slope are studied.

## Selection of slope geometric parameters

With the help of Midas GTS NX finite element software for numerical simulation analysis, Midas GTS NX is a general finite element analysis software developed for geotechnical fields in Midas series software. The software supports static analysis, dynamic analysis, seepage analysis, stress-seepage coupling analysis, consolidation analysis, construction stage analysis, slope stability analysis, and other types of analysis.

Topography is an important geological condition. The type and spatial distribution of geological disasters are largely controlled by the characteristics of topography, spatial distribution, and their mutual combinations^30^. According to the characteristics of the geometric parameters of the oblique-cut rock slope in the western region of Henan Province, a numerical model of the rock slope has been established. The slope type is set to be linear, and the variables are the slope height and slope gradient.

According to the survey results of landslide disasters in the western region of Henan Province and the previous statistical experience^31^, slope disasters are more likely to occur on slopes with a relative elevation of 40–60 m. Therefore, the slope height interval of the slope model is set to 40–60 m. In order to facilitate the calculation, the value is taken every 5 m from 40 m, and a total of 5 slope model heights are selected. The main development slope range of a landslide is 45°–60°, so the slope range of the slope model is set to 45°–60°, and the value is taken every 5° from 45°, and a total of 4 slope models are set. Various types of slope geometric parameters are shown in Table 1.

**Table 1:** Geometric parameters for rock slope.

Gradient	Height				
	40 m	45 m	50 m	55 m	60 m
45°	Type 1	Type 2	Type 3	Type 4	Type 5
50°	Type 6	Type 7	Type 8	Type 9	Type 10
55°	Type 11	Type 12	Type 13	Type 14	Type 15
60°	Type 16	Type 17	Type 18	Type 19	Type 20

## Slope locking section setting

Due to the different lithology of the stratum, the number of geological disasters developed on the surface has obvious distribution differences. The structure and occurrence of the rock mass directly affect the formation and development of the landslide^32^. In terms of lithology, the rock landslide disasters in the western part of Henan Province are mainly developed in sandstone, shale, and mudstone. Soft rock, low-strength rock mass, or soft-hard interbedded rock mass are the main disaster-pregnant rock masses. In the western part of Henan Province, the lithology of the oblique-cut locking rock slope is mainly composed of medium-high-strength rock masses such as sand-mudstone and sandstone, which tend to the outside of the slope, and the dip angle is 20°–40°. The slope is layered, but it does not have the conditions to slide out along the stratum. The slope can only slide out of the stratum at a certain angle. The upper part of the slope slides along the time-dependent deformation of the stratum, and the trailing edge is cracked. There is a weak interlayer at the foot of the slope that acts as a sliding zone. The upper part of the weak interlayer is the unconnected area of the potential sliding surface, which is the locking section of the slope.

Therefore, in the numerical model, the lithology of the formation is determined to be mainly sandstone, the weak interlayer is mudstone, and the inclination angle of the mudstone layer is 20°–40°. The locking section connected to the mudstone layer plays a controlling role in slope stability. The inclination angle of the mudstone layer is taken once every 5° from 20°, and a total of 5 inclination angles are set. In order to restore the characteristics of vertical joint fissure development in landslides characteristics, vertical tensile cracks are set at the trailing edge of the slope.

**Figure 2:**
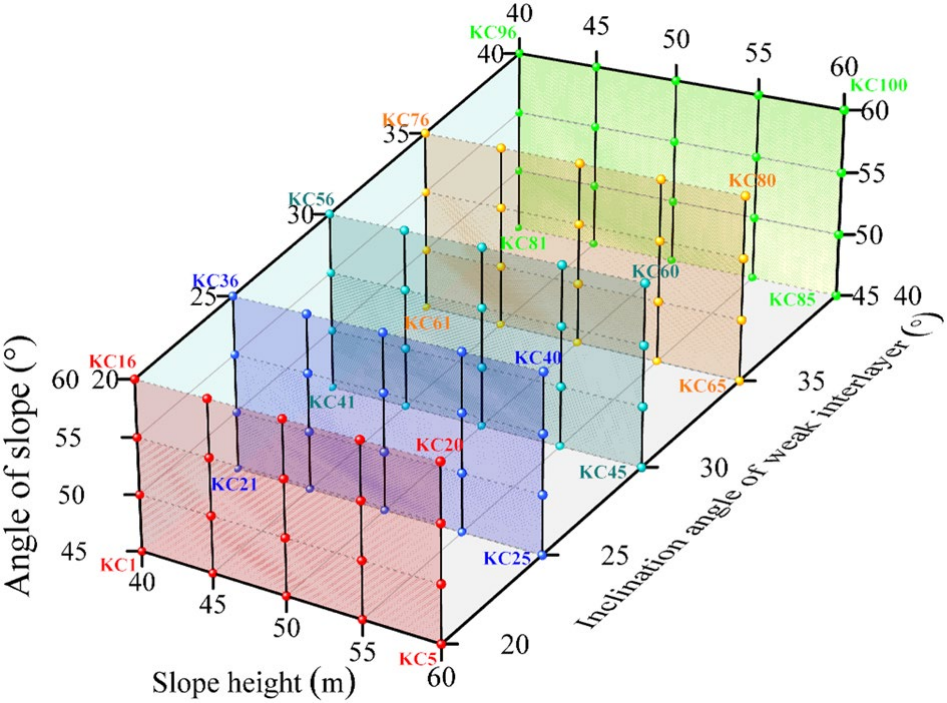
The parameter display diagram of each calculation condition.

Under the influence of the superposition of three variables: slope gradient *α*, slope height *H*, and rock dip angle *β*, after permutation and combination, a variety of simulation calculation conditions are set in order, and the calculation parameters of each working condition are characterized by (*α*,*H*,*β*).With ‘KC’ as the code, a total of 100 simulation schemes are set in order. The model calculation parameters of each working condition are shown in Fig. 2. Among them, the positive direction of X axis represents the increase of slope height, the positive direction of Y axis represents the increase of inclination angle of weak interlayer, and the positive direction of Z axis represents the increase of slope. The 20 working conditions with the same inclination angle of weak interlayer are marked with the same color.

**Figure 3:**
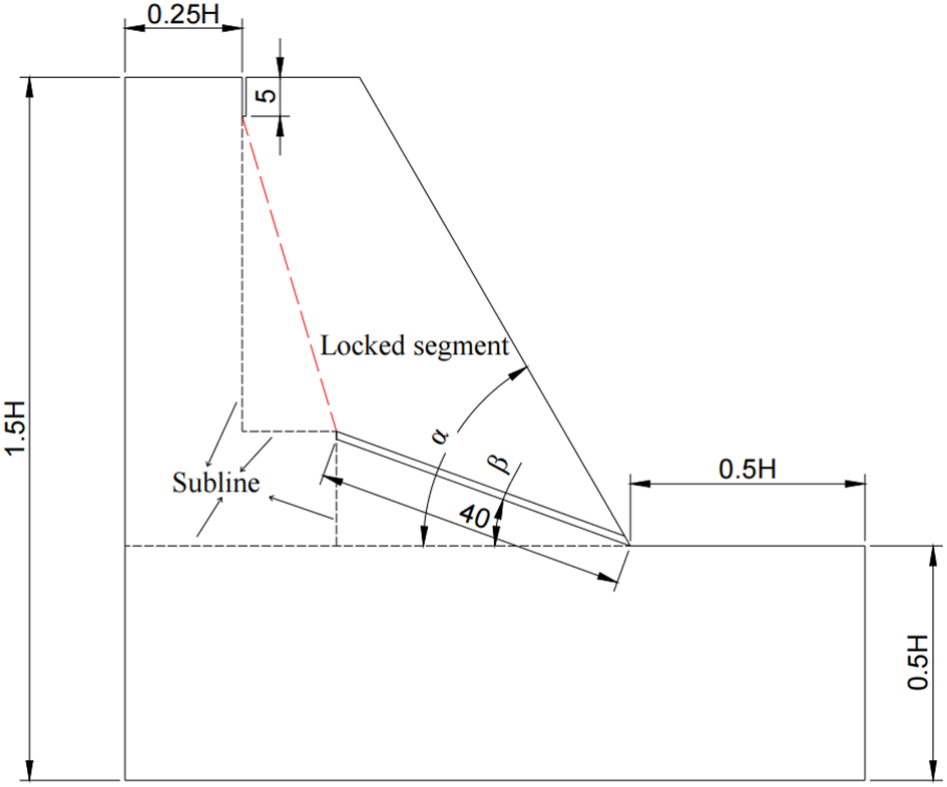
Schematic diagram of numerical model locking section.

For the locked rock slope, the range of the weak interlayer will change according to the dip angle of the rock stratum. Changing the length of the weak interlayer and the geometric parameters of the slope is equivalent to changing the length and position of the locked section of the slope (Fig. 3).

In this paper, the length of the weak interlayer is fixed. Before the numerical simulation, using the auxiliary line design calculation formula (1) in Fig. 3. This formula can be substituted into the slope geometric parameters of each working condition to estimate the potential locking section length (red dotted line) in all working conditions, so as to compare and verify after the simulation calculation.

1 $$ L_{s} = \sqrt {\left[ {\left( {H - 5 - 40 \times {\text{Sin}} \left( \beta \right)} \right)} \right]^{2} + \left[ {0.25 \times H + \frac{H}{{{\text{Tan}} \left( \alpha \right)}} - 40 \times {\text{Cos}} \left( \beta \right)} \right]^{2} } $$

**Figure 4:**
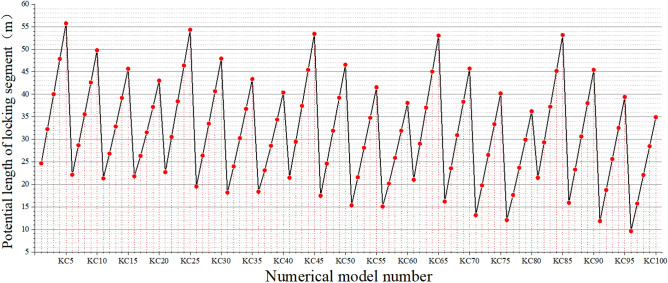
Calculation result chart of potential locking section length.

In the formula, *L*_*s*_ is the length of the potential locking segment; *H* is the slope height; *α* is the slope angle; *β* is the dip angle of the weak interlayer. The length of the potential locking section calculated under different working conditions is shown in Fig. 4:

Comparing Fig. 2 with Fig. 4, the slope calculation model starts from KC1, and the slope height changes first. Every 5 groups are a change period. When the slope height reaches the peak (60 m), it is restored to 40 m, and the slope increases by 5°. Continue to change the slope height from low to high, and so on, until the slope and slope height reach the maximum value, increase the 5° weak interlayer dip angle, and other parameters begin to change again. Therefore, every 5 sets of calculation conditions are a small change period, and every 20 sets of calculation conditions are a large change period. The length of the potential locking section of the slope is determined by the slope height, the slope angle and the dip angle of the weak interlayer. The dip angle of the weak interlayer controls the length of the potential locking section at the large period level. With the increase of the dip angle of the weak interlayer, the overall length of the potential locking section decreases. In contrast, slope height and slope angle control the length of potential locking section in a small period. When the slope height increases, the length of potential locking section increases linearly (such as KC1–KC5, KC6–KC10, etc.). The influence of slope angle on the length of potential locking section is not linear. With the increase of slope angle, the length of potential locking section decreases first and then increases (such as KC1 and KC6, KC2 and KC7, etc.).

**Figure 5:**
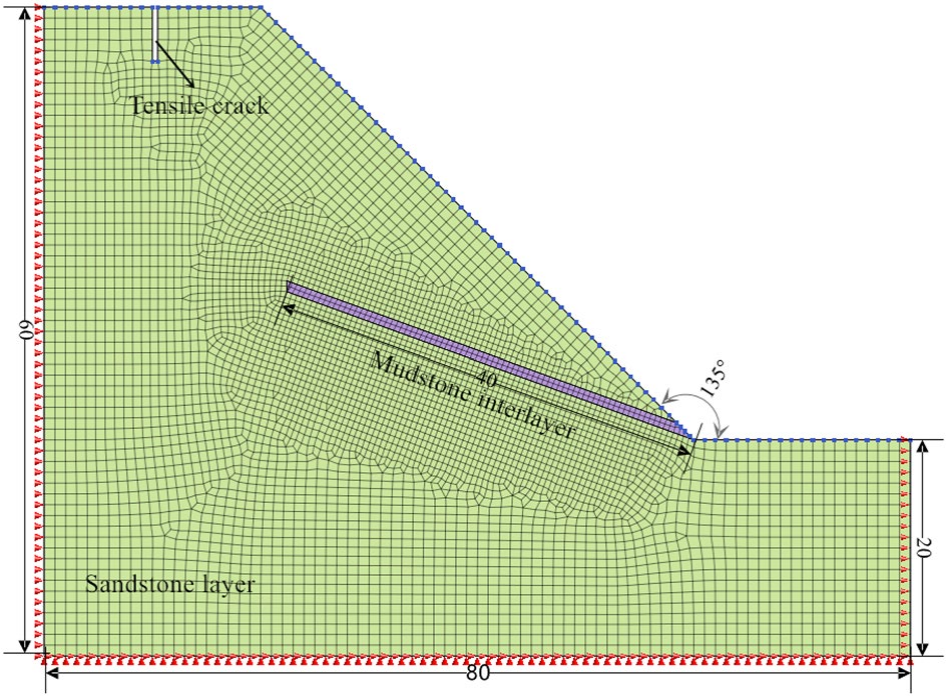
Cross layer oblique cut locking rock slope KC1.

Taking working condition 1 of an oblique-cut locking rock slope as an example (Fig. 5), the total length of the model is 80 m, the total height is 60 m, the slope gradient is 45°, the main lithology is sandstone, and there is a mudstone interlayer at the foot of the slope. The dip angle is 20°, the thickness is 2 m, the length is 40 m, and The tensile cracks at the trailing edge have a great influence on the stability of the slope. Combined with the survey results of Li et al.^31^ on the cracks at the trailing edge of the landslide in the western part of Henan Province, the cracks in the generalized model are set to be 5 m deep and 1 m wide. The physical and mechanical parameters of rock mass are obtained by the TAW-2000 microcomputer-controlled rock triaxial test system. The instrument can carry out uniaxial compression, conventional triaxial, and shear tests of rock, and the test results are reliable. The basic physical and mechanical parameters of rock are measured by a standard cylinder (diameter 50 mm, height 100 mm). The uniaxial compression test is carried out by the displacement loading control method. The loading rate is set to 0.1 mm/min. The direct shear test is carried out according to the requirements of the test procedure. The normal stress is loaded in three stages, and the horizontal shear force is controlled by displacement loading. The loading rate is the same as that of the uniaxial compression test. Through the above laboratory tests and previous experience^33^, the rock mass parameters are obtained as shown in Table 2.

**Table 2:** Strength parameters of rock.

Rock type	Natural unit weight (kN/m^3^)	Saturated unit weight (kN/m^3^)	E (MPa)	μ	Φ (°)	C (kPa)	K (m/h)
Sandstone	21.1	22.3	375	0.28	33	65	0.035
Mudstone	18.7	19.6	75	0.35	25	35	0.0018

Using Midas GTS NX for finite element simulation, a two-dimensional model is established, as shown in Fig. 5. The numerical calculation of the Midas GTS NX is based on the strength reduction method. The core is to reduce the strength parameters of rock and soil in equal proportion until the strength parameters after reduction make the slope reach the critical state. In order to simplify the calculation and highlight the regularity, the sandstone layer is set to a single layer. The model has a total of 5333 nodes and a total of 5221 elements. The element type is a two-dimensional plane strain element. The rock constitutive model adopts the Mohr–Coulomb model, and the sandstone and the weak interlayer are set to be in direct contact. The model mesh is divided by a hybrid mesh generator. The green mesh represents the sandstone, and the purple mesh represents the weak interlayer. The boundary condition of the model is the horizontal displacement of the left and right boundary constraints, and the bottom is a fixed constraint. In addition, the seepage surface of the slope is set on the slope surface, so that rainfall can only penetrate into the slope from the slope surface and cannot cross the boundary.

As a kind of soft rock that is easy to disintegrate in water, the permeability parameters of sandstone have a great influence on slope stability under rainfall conditions. Due to the existence of an unsaturated zone, rainfall infiltration leads to a decrease in soil suction, which in turn affects the shear strength of the soil. The description of the unsaturated soil infiltration process requires the soil water characteristic curve (SWCC) and permeability function. For the unsaturated characteristics of soil, Alfrendo Satyanaga and other scholars have conducted in-depth research^34,35^. Considering the unsaturated characteristics of soil, this is very necessary. The VG model is a model of the unsaturated characteristic function curve proposed by Van Genuchten in 1980^36^. It has been widely used in groundwater analysis, seepage analysis, and various software. The expression of the VG model is shown in Formulas (2) and (3):

2 $$ \theta = \theta_{r} + \frac{{\theta_{s} - \theta_{r} }}{{(1 + \left| {ah} \right|^{n} )^{m} }} $$

3 $$K(h) = K_{s} \frac{{\left\{ {{\text{1}} - \left( {ah} \right)^{{n\quad 1}} \left[ {1 + \left( {ah} \right)^{n} } \right]^{m} } \right\}^{2} }}{{({\text{1}} + \left| {ah} \right|^{n} )^{{m/n}} }}$$

In the formula: $m = 1 - \frac{1}{n}$; $\theta_{r}$ is the residual water content; $\theta_{s}$ is saturated water content; *h* is negative pore water pressure; *a*, *n*, *m* is the shape parameter of the unsaturated characteristic function curve; $K_{s}$ is the saturated permeability coefficient; *K*(*h*) is the unsaturated permeability coefficient.

**Figure 6:**
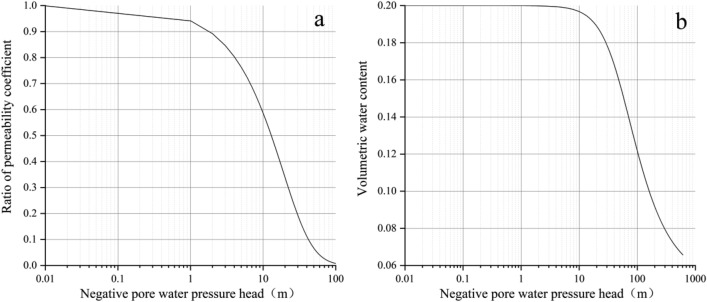
Unsaturated characteristic result diagram: (**a**) permeability coefficient function; (**b**) volume water content function.

The saturated permeability coefficient of sandstone is 0.035 m/h, and the saturated volumetric water content is 0.2. In this paper, the unsaturated permeability coefficient of sandstone is simulated based on a VG model combined with finite element software, and the results are shown in Fig. 6.

## Rainfall condition setting

**Figure 7:**
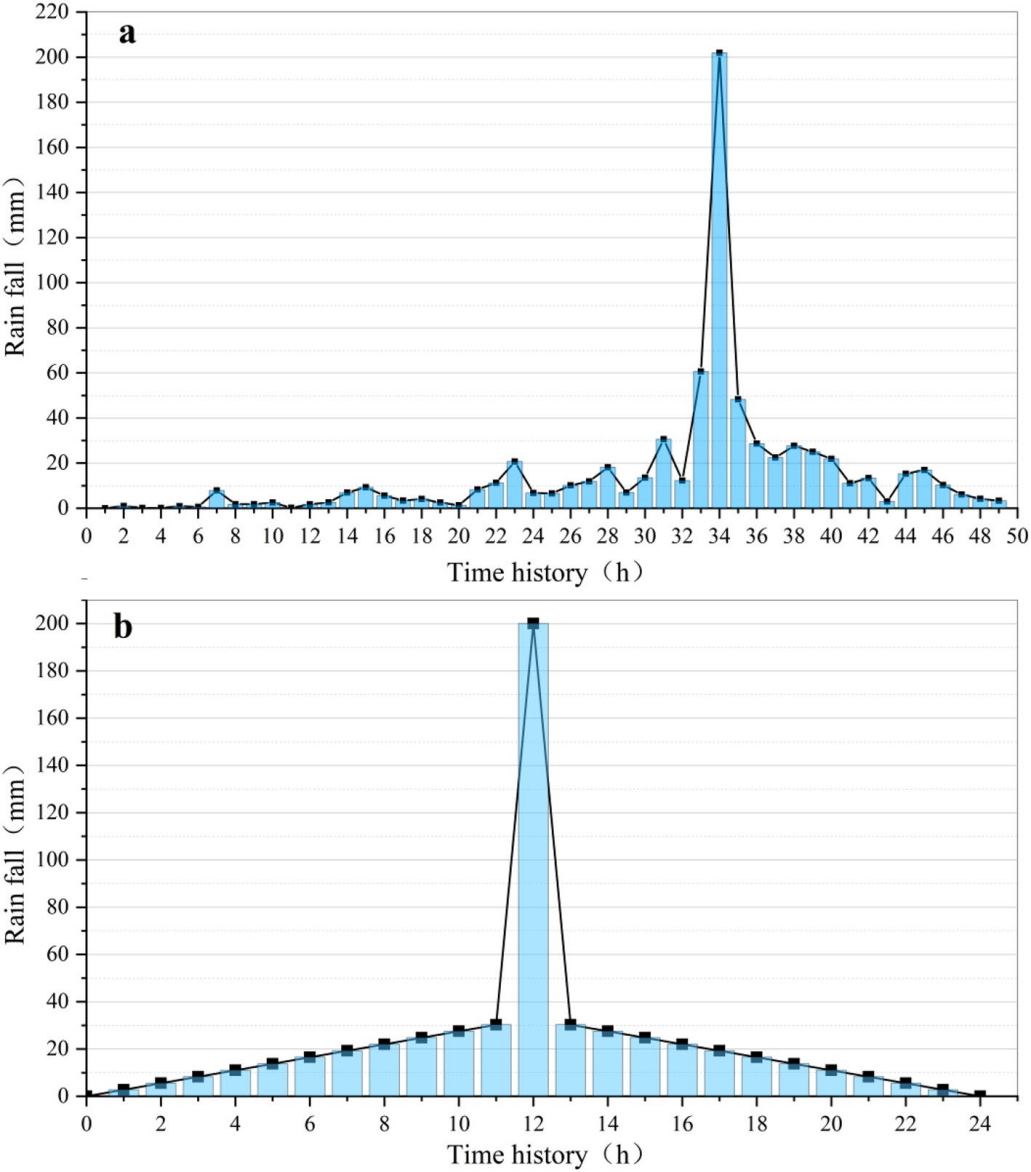
Rainfall history curve.

In order to study the dynamic response mechanism of the oblique-cut locked rock slope under the influence of such heavy rainfall in the western part of Henan Province, this paper takes the rainfall type and rainfall of Zhengzhou ‘7·20’ as a reference^16^ (Fig. 7a) and sets the rainfall conditions of this fluid–solid coupling simulation analysis (Fig. 7b): The rainfall type is the center type, the duration is 24 h, the rainfall intensity suddenly changes to 200 mm/h at the beginning of the 12th hour, and the rest of the time is linear, and the total rainfall of the simulated 24 h is generally consistent with the total rainfall of the actual case, about 640 mm. The increase in rainfall intensity per hour of simulated rainfall is calculated to be 2.75 mm/h.

## Deformation characteristics and instability mode of oblique-cut locking rock slope

**Figure 8:**
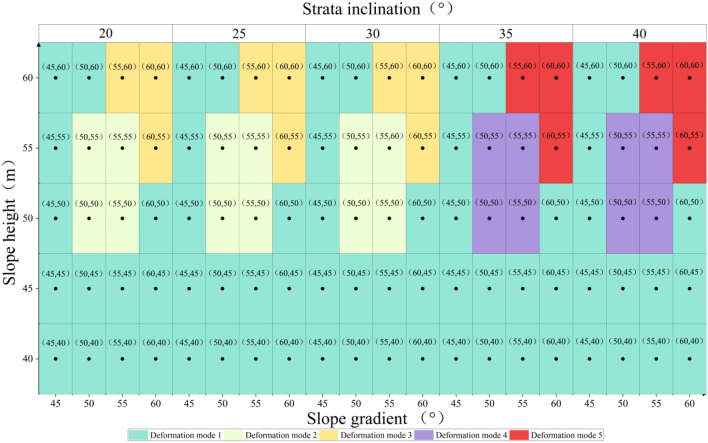
Distribution map of slope deformation and failure modes.

The numerical simulation calculation is based on the change of slope toe *α*, slope height *H*, and weak interlayer dip angle *β*, and is displayed in 100 calculation conditions. According to the different deformation and failure characteristics of the slope during the calculation of the strength reduction method, five typical instability failure modes of the cross-layer oblique-cut locking rock slope in western Henan are summarized and proposed (Fig. 8).

### The slip shear failure along the bedding plane at the toe of the slope

**Figure 9:**
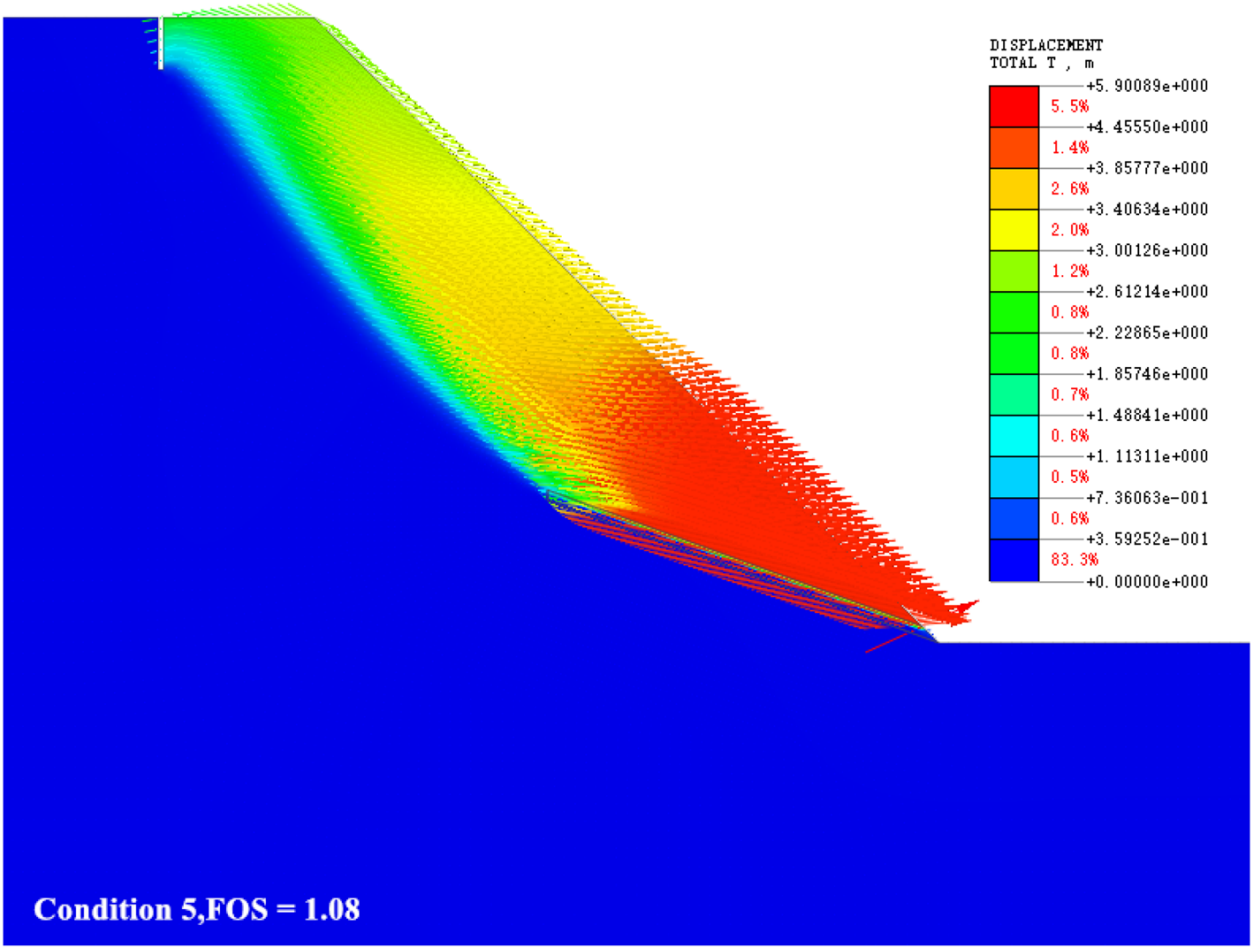
The slip shear failure along the bedding plane at the toe of the slope.

As shown in Fig. 9, when the slope gradient and slope height are small (generally slope < 50°, slope height < 50 m), the slope is affected by its own gravity to produce slip force along the weak interlayer structural plane, and stress concentration is formed at the foot of the slope. The deformation and failure of the bottom of the slope are earlier than those of the top of the slope. The bottom of the slope shows a slip movement trend along the rock structure towards the free surface of the slope, resulting in slip-shear deformation. The static and dynamic water pressure formed by heavy rainfall infiltration softens the sandstone and mudstone layers. When the strength of the structural plane of the rock layer is reduced to a certain extent, the intersection between the tensile cracks at the trailing edge of the slope and the weak interlayer becomes a potential sliding surface, which controls the overall stability of the slope, that is, the locking section, which bears the function of resisting the sliding force. As the slope continues to deform, the trailing edge cracks deeper. Under the combined action of shear failure and rainfall infiltration, when the locking section of the slope is not enough to resist the sliding force, the locking section of the slope is destroyed, and the sliding body rushes out from the slope toe along the level to the free surface to form an overall sliding-shear failure.

### The slip shear failure along the bedding plane at the toe of the slope (the sliding surface moves up)

**Figure 10:**
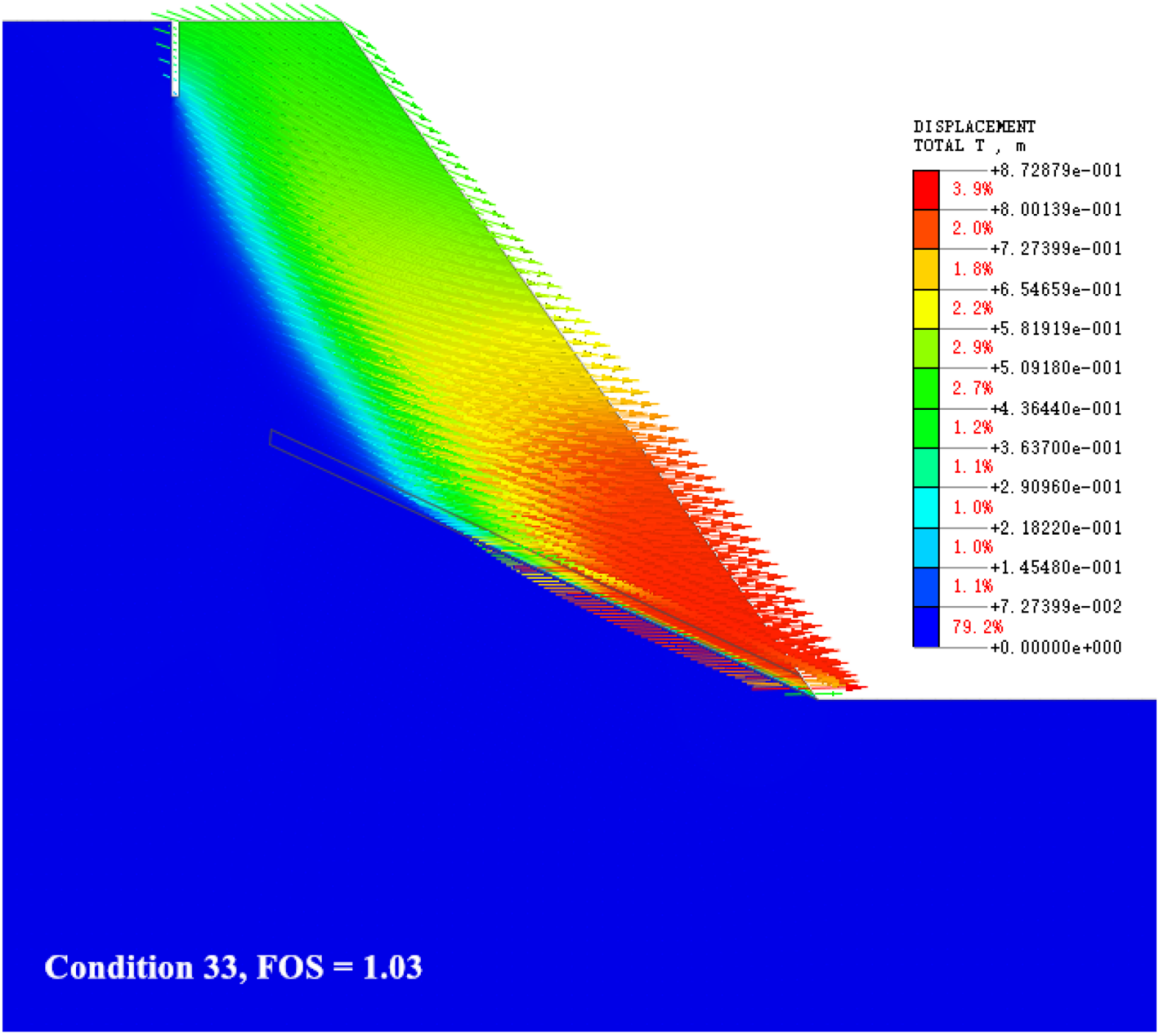
The slip shear failure along the bedding plane at the toe of the slope (the sliding surface moves up).

As shown in Fig. 10, with the increase of the geometric parameters of the slope (the slope is 50°–55° and the slope height is 50–55 m), the shape of the slope becomes high and steep, and the failure mode of the slope is still dominated by the slip shear failure along the slope toe. Due to the difference in the ability of the weak interlayer in the slope and the sandstone contacted up and down, the distribution and mechanical model of tectonic stress are different^37^. The deformation area inside the slope moves upward from the trailing edge of the weak interlayer and gradually rises. The upper edge of the rock stratum shows a tendency to move to the free surface of the slope under the action of lateral pressure and gradually bends and cracks. The potential sliding surface of the previous stage is no longer used as the best failure path, and the range of the locking section that controls the stability of the slope changes and develops outside the slope.

### The shear failure in the middle of the slope surface

**Figure 11:**
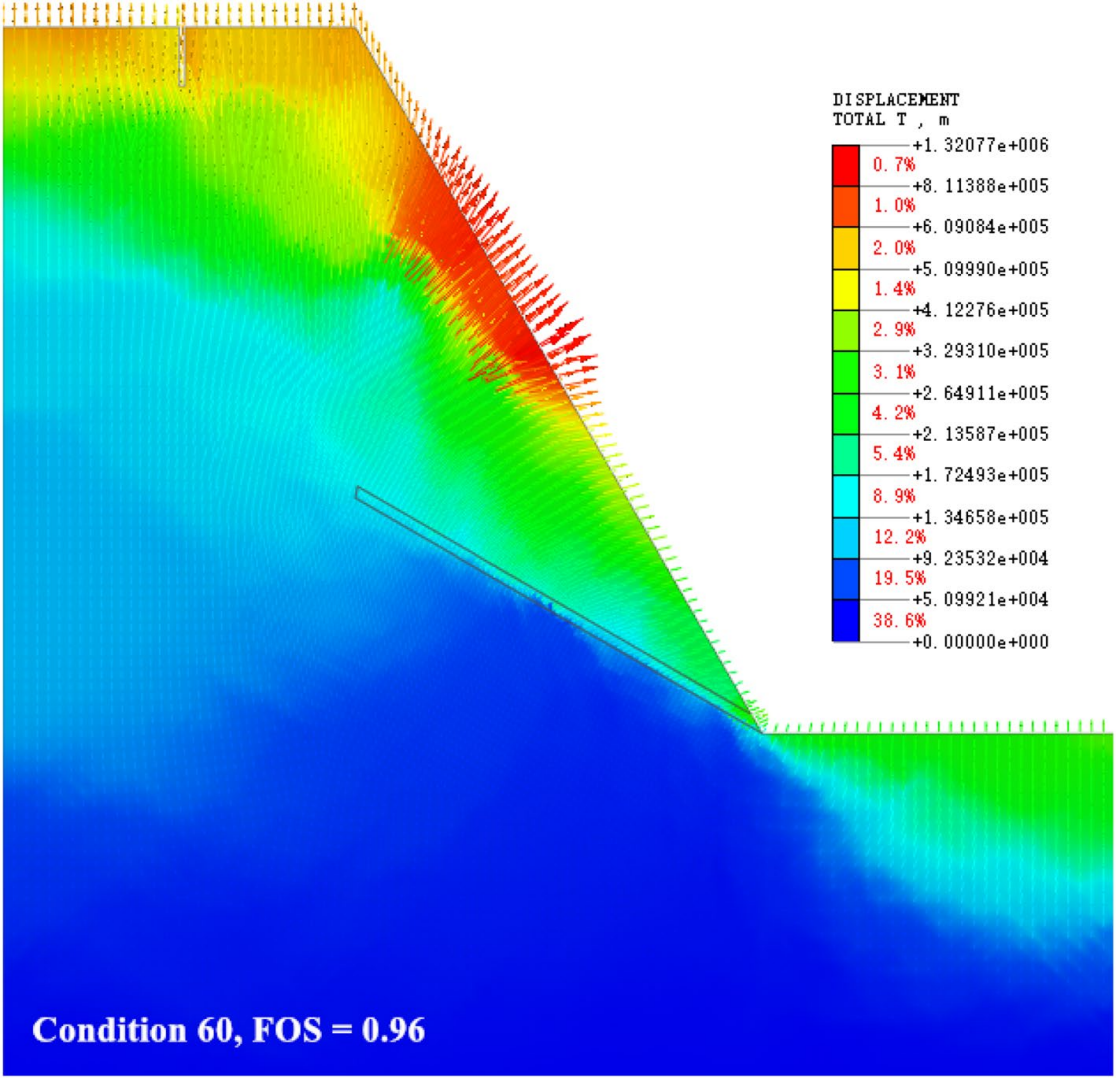
The shear failure in the middle of the slope surface.

As shown in Fig. 11, when the slope gradient and slope height reach the peak (the slope is 55°–60° and the slope height is 55–60 m), but the dip angle of the weak interlayer is small, the internal deformation concentration area of the slope continues to move up. The slope shows a sliding shear failure mode in which the slope top develops along the structural surface to the middle of the slope, and a large range of deformation signs appear in the deep part of the slope. Under this failure mode, the overall deformation area of the slope becomes larger, the anti-sliding force on the slope structural surface is less than the sliding force along the structural surface caused by self-gravity, and the failure of the slope top and slope surface takes precedence over the slope toe. With the continuous development of deformation, the main sliding surface is penetrated, the locking section is broken, and the rock mass on the sliding surface slides out along the main sliding surface.

### The slip shear failure along the bedding plane at the toe of the slope (the sliding surface moves down)

**Figure 12:**
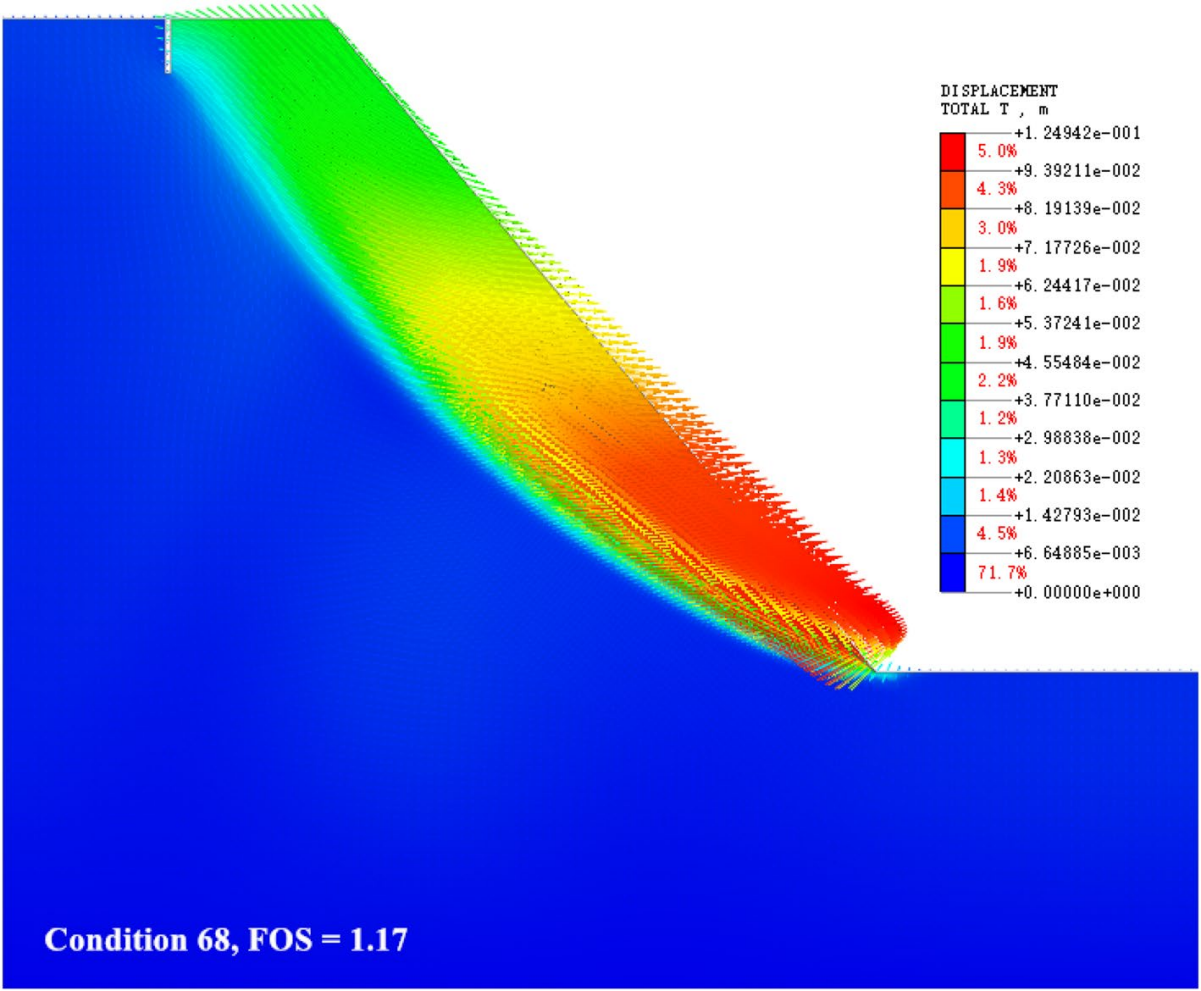
The slip shear failure along the bedding plane at the toe of the slope (the sliding surface moves down).

As shown in Fig. 12, the slope gradient and slope height are high, and the dip angle of the weak interlayer gradually increases (> 30°). The failure mode of the slope is restored to the slip shear failure along the slope toe. Under the action of heavy rainfall, rainwater infiltrates along the cleavage surface of sandstone, forming a continuous softening of the weak interlayer of mudstone. The softening effect of water makes the mudstone layer gradually evolve into a muddy interlayer, and the strength is reduced^38^. Due to the increase in the dip angle of the weak interlayer, the spatial position inside the slope gradually approaches the slope surface, which leads to an advance in the contact time of the weak interlayer with rainwater and an extension of the seepage time. In addition, the weak interlayer can tolerate a large amount of deformation, and with the development of deformation, the sliding body structure is obviously loosened, the rainfall infiltration intensity is increased, the slope deformation range is further increased, and the potential sliding surface is developed to the depths.

### The uplift shear failure at the bottom of the slope

**Figure 13:**
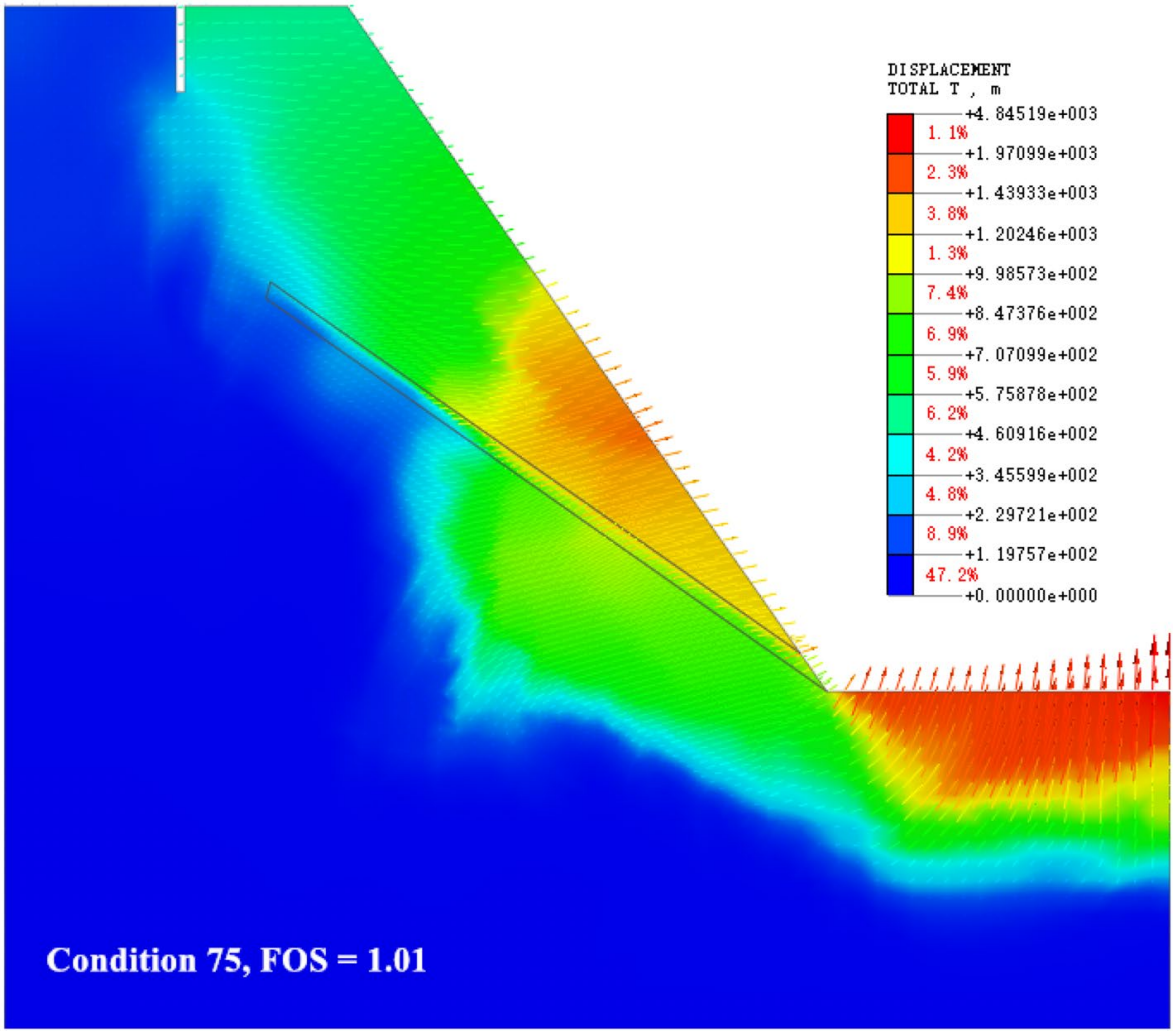
The uplift shear failure at the bottom of the slope.

Figure 13 shows the uplift shear failure occurred at the bottom of the slope. Because the dip angle B of the weak interlayer is much larger than the internal friction angle of the rock structure surface, it becomes the dominant factor. The slope rock stratum will slide downward along the bedding plane, resulting in deformation accumulation along the rock stratum direction. Long-term gravity and lateral pressure make the lower edge of the rock stratum uplift and bend to the free surface. Stress concentration occurs at the lower edge of the slope rock stratum. Especially when the rock stratum is weak, the bending deformation will be more obvious, and even buckling deformation and failure will occur. At the same time, the middle of the slope shows obvious interlayer dislocation, forming tensile cracks along the bedding plane. At this time, the rock stratum slides downward along the bedding plane under the action of gravity to produce shear displacement, and at the same time, normal displacement is generated under the action of lateral pressure and the self-weight of the rock stratum.

## Analysis of stability variation law of oblique-cut locking rock slope

**Figure 14:**
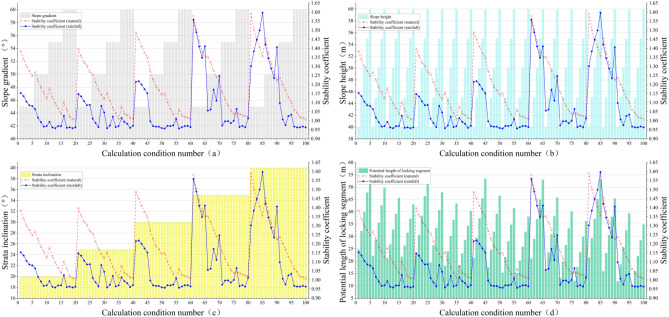
The relationship between different influencing factors and slope stability coefficient: (**a**) slope angle; (**b**) slope height; (**c**) weak interlayer inclination angle; (**d**) potential length of locking segment.

Using the strength reduction method, the stability coefficients of the slope before and after rainfall under different slope-influencing factors can be obtained. The results are shown in Fig. 14. This figure reveals the relationship between the stability coefficient of the slope, the slope angle, the slope height, the dip angle of the weak interlayer, and the length of the potential locking section. The slope gradient under the simulation calculation condition is set to 5° as the change interval, increasing step by step, and every 20 groups of slope models are a cycle. The slope height takes five slope types as a change cycle, and every five groups of slope models are a cycle. The inclination angle of the weak interlayer is 5° as the change gradient, and the parameter is consistent in each of the 20 groups of slope models. The length of the potential locking section has been described above, and the length of the potential locking section under different working conditions is not the same.

**Figure 15:**
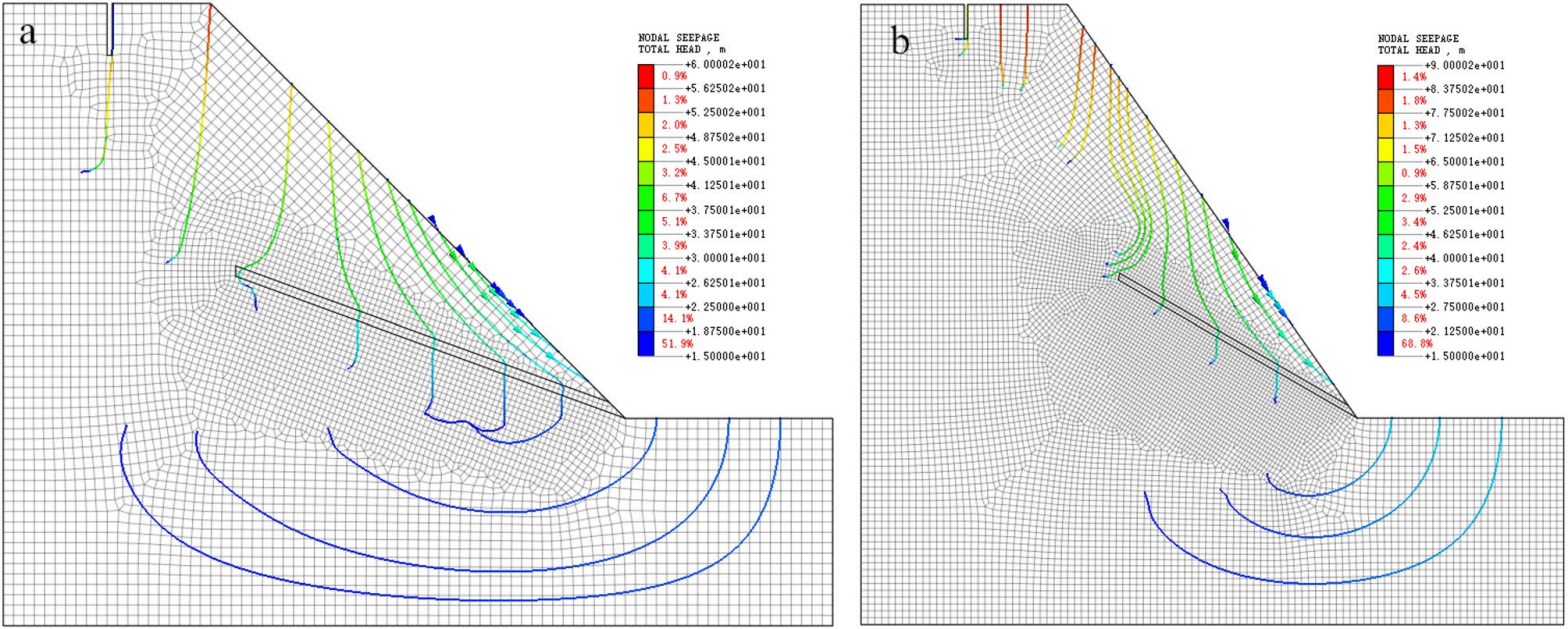
Rainfall infiltration and seepage flow through: (**a**) due to the smaller size of the slope; (**b**) when the slope size is large.

In terms of slope geometric factors, it can be seen from Fig. 14a, b that when the inclination angle of the weak interlayer is constant, as the slope gradient and slope height gradually increase, the high and steep slope type leads to an increase in the self-weight stress of the slope. At the same time, the anti-sliding force is reduced, and the slope stability is gradually reduced. The geometric factors of the slope control the overall trend of the slope stability coefficient. When the slope is low (< 45°), the slope stability coefficient decreases slowly with the increase in slope height. When the slope is higher (> 45°), the stability coefficient of the slope drops sharply. With the arrival of the new change cycle, the slope height decreases; even if the slope is higher, the slope stability will be slightly improved, but it is also in a near-instable state. In addition, as shown in Fig. 15a, the infiltration of extreme heavy rainfall leads to a rapid increase in groundwater level inside the slope, and the direction of the seepage flow path is outside the slope. The types of influence on the slope are divided into hydrostatic pressure effects, uplift force effects, saturated softening effects of sliding zones, and seepage pressure effects^39^. Due to the particularity of the bedding rock slope and the weak interlayer, the role of groundwater in this type of landslide is mainly the drag force, the uplift pressure on the sliding surface of the slope, and the fissure water pressure at the trailing edge of the slope^40^. Therefore, the stress composition of the slope body is greatly changed, resulting in a sharp decrease in the stability of the slope compared with the natural state, and the number of unstable slopes is increased.

It can be seen from Fig. 14c,d, and the failure mode of the slope that the dip angle of the weak interlayer has an obvious influence on the stability coefficient of the slope. With the gradual increase of the dip angle of the weak interlayer, the failure mechanism is basically summarized as follows: the slip shear failure along the bedding plane at the toe of the slope → the sliding surface moves up → the shear failure in the middle of the slope surface → the sliding surface moves down → the uplift shear failure at the bottom of the slope. The position of the potential dangerous failure surface of the slope under different weak interlayer dip angles is quite different. When the geometric factor parameters of the slope are small and the dip angle of the weak interlayer is less than 30°, as shown in Fig. 15b, most of the seepage flow path points to the slope. The drag force on the sliding surface of the slope changes with the direction of seepage; the potential sliding surface moves upward, the volume of the sliding body decreases, and the sliding force generated by the self-weight at the sliding surface decreases. Constrained by the rock mass of the locking section, the length of the potential locking section increases, and the slope stability coefficient increases. The length of the potential locking section in individual conditions (such as conditions 65 and 85) exceeds 50 m. Under the superposition of factors such as the change in seepage path and the small size of the slope, the stability coefficient increases after rainfall. When the inclination angle of the weak interlayer is greater than 30°, the weak interlayer is close to the slope surface, the area of the tensile failure zone increases, and the potential sliding surface turns to deep development. At this time, the range of the slope locking section increases, the length of the potential locking section increases, the sliding force is not enough to support the penetration of the locking section, and the slope stability coefficient is further improved. When the geometric factors of the slope are large (slope > 55°, slope height > 55 m), the self-weight stress of the slope increases sharply, and the sliding thrust of the locking section is increasing, which causes the stress concentration at the interface between the locking section and the sliding bed. The degree is getting higher and higher, and the control effect of the locking section on the stability of the landslide is getting stronger and stronger. Once the shear stress of the locking section exceeds its shear strength, the overall instability of the sliding body occurs in a very short period of time, which is also the reason for the slope instability in the final stage of each parameter change cycle in the simulated working condition.

## Case analysis of actual landslide

**Figure 16:**
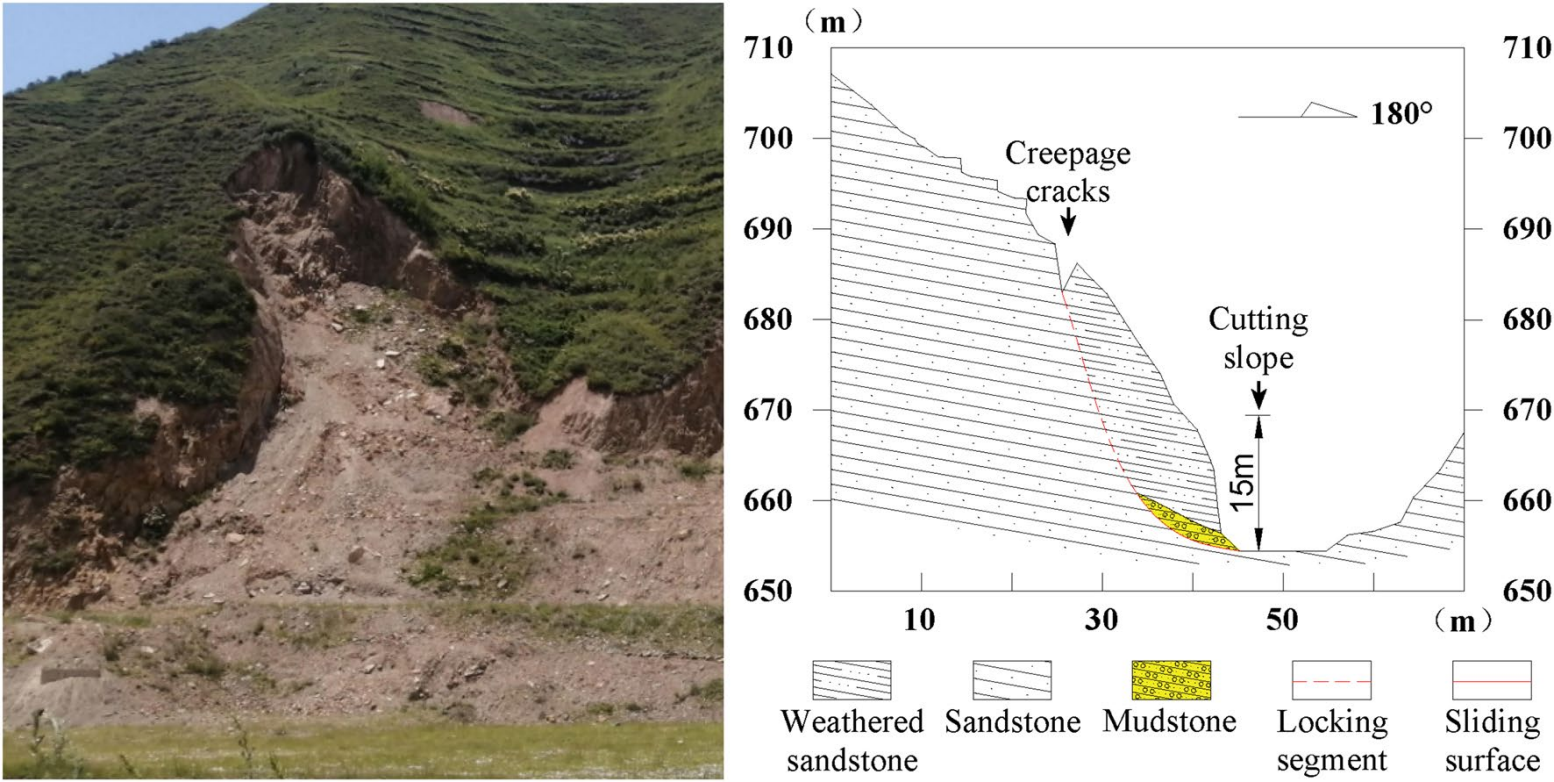
Landslide and its profile map in Huaxi Village, Santun Township.

**Figure 17:**
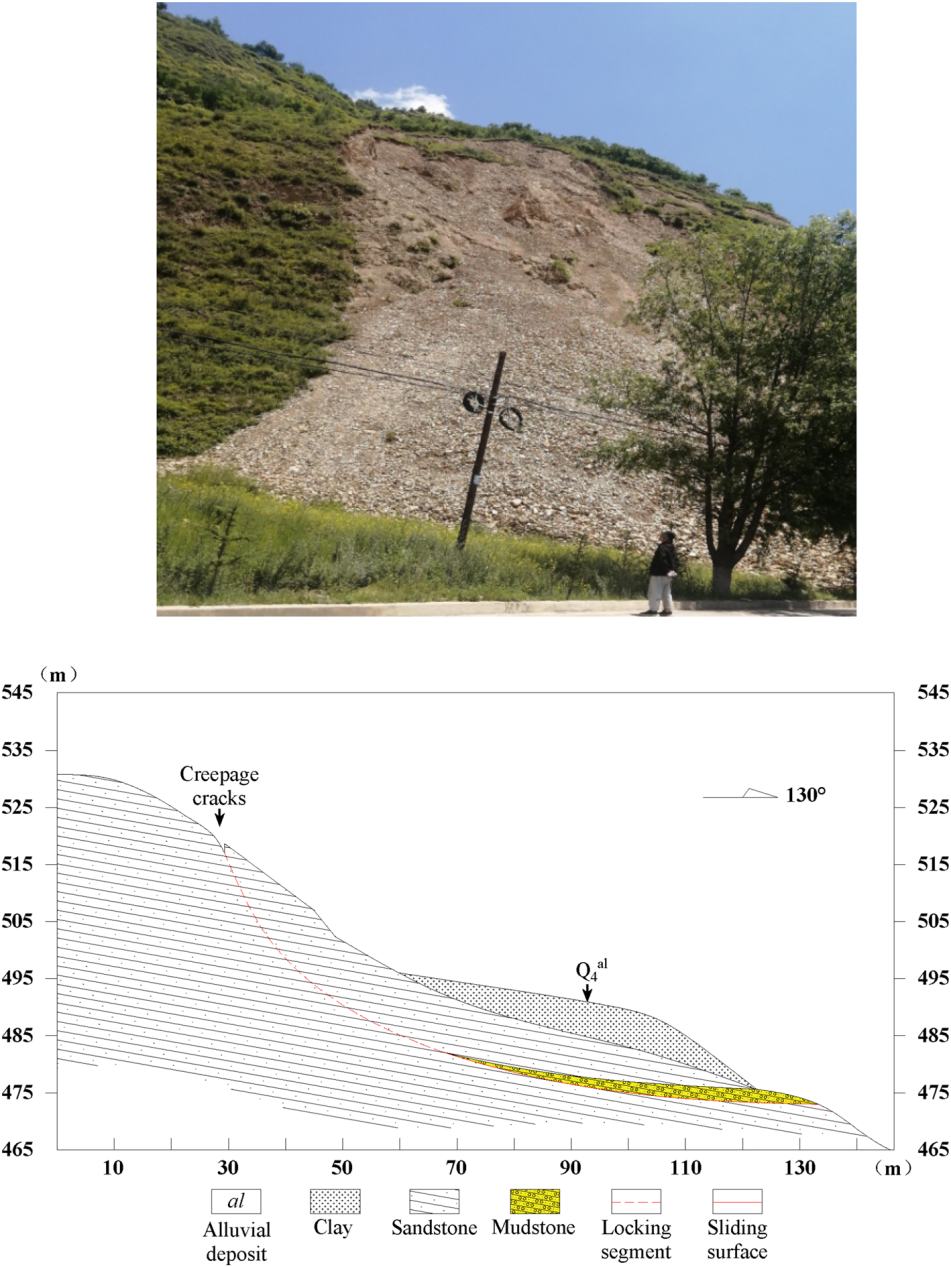
Lijiazhuang Landslide and Its Profile in Shaoyuan Town.

The oblique-cut locked rock slope is layered, but it does not have the conditions to slide out along the stratum level^15^. For example, the slope can only slide out from the stratum at a certain angle when the reverse slope or the dip angle of the stratum is greater than the slope angle. Before the cut-through part is penetrated, it can be regarded as the locked section and the carrier of stress concentration. In view of the fact that the potential macroscopic fracture transfixion surface of the locking section intersects with the stratum level at a certain angle, it is called ‘the oblique cutting type’. The Huaxi Village landslide in Santun Township, Ruyang County, Henan Province, and the Lijiazhuang landslide in Shaoyuan Town are the representatives of this type of locked slope (Figs. 16 and 17).

(1) The Huaxi Village landslide in Santun Township is located in a low mountainous area with an altitude of 650–700 m. The exposed rock mass is Mesoproterozoic sandstone with an attitude of 150° ∠40°. The weathering degree of the surface rock layer is high, which is mixed with the Quaternary Middle Pleistocene residual slope brown-red clay. The slope aspect is 190°, the slope is 65°, and the slope height is 55 m. The landslide is a rock landslide that first occurred in August 2002. It slid again on July 20, 2021, and formed an arc-shaped crack with a length of 45 m, a width of 2.7 m, and a depth of 3.7–6.5 m at the trailing edge of the landslide. The cracks at the trailing edge of the landslide have an obvious ring chair shape and have developed into a landslide sliding wall. The average height of the sliding wall is 2.2 m. The length of the sliding body is 28 m, the width is 45 m, the average thickness is 2.0 m, and the scale is 2520 m^3^.

The lower part of the slope is cut by an artificial slope, resulting in a steep slope angle and expanding the free surface; secondly, the infiltration of rainfall increases the water absorption bulk density of weathered sandstone, which makes the friction coefficient between the water-bearing rock soil and the lower non-water-bearing rock soil smaller and the anti-sliding force lower. Due to the traction force formed by the increase in self-weight, arc cracks begin to appear on the slope, cracks continue to increase, sliding surfaces run through, and landslides occur.

(2) The Lijiazhuang landslide in Shaoyuan Town is located on the east side of the Lijiazhuang Highway in Shaoyuan Town, Jiyuan City. The landslide is 150 m long and 200 m wide. The landslide is a rock landslide. The sliding body material is Permian strongly weathered mudstone, overlying Quaternary slope pluvial deposits, and there are arc cracks with a depth of 1.6–3.5 m at the trailing edge. According to the analysis of geophysical data, the sliding surface of the landslide is distributed in the third layer of mudstone. The overlying strata are silty clay and strongly weathered sandstone, with an average thickness of 8 m. The sliding bed is the weakly weathered mudstone in the lower part.

The landslide is located in the slope zone; the trailing edge of the topographic slope is greater than 30°; and the leading edge of the landslide is a valley, forming a free surface. The villagers build houses and cut slopes, which destroys the stability of the slope. Under the action of gravity, it provides potential energy and a free surface for the sliding body. The sliding body remains stable in its natural state. When there is heavy rainfall, the mudstone layer softens rapidly in its saturated state, forming a weak structural plane. The surface water infiltrates into the weak structural plane to form a saturated zone, thereby reducing the mechanical parameters of the sliding surface soil layer. When the locking section reaches the bearing capacity limit, a landslide occurs.

**Figure 18:**
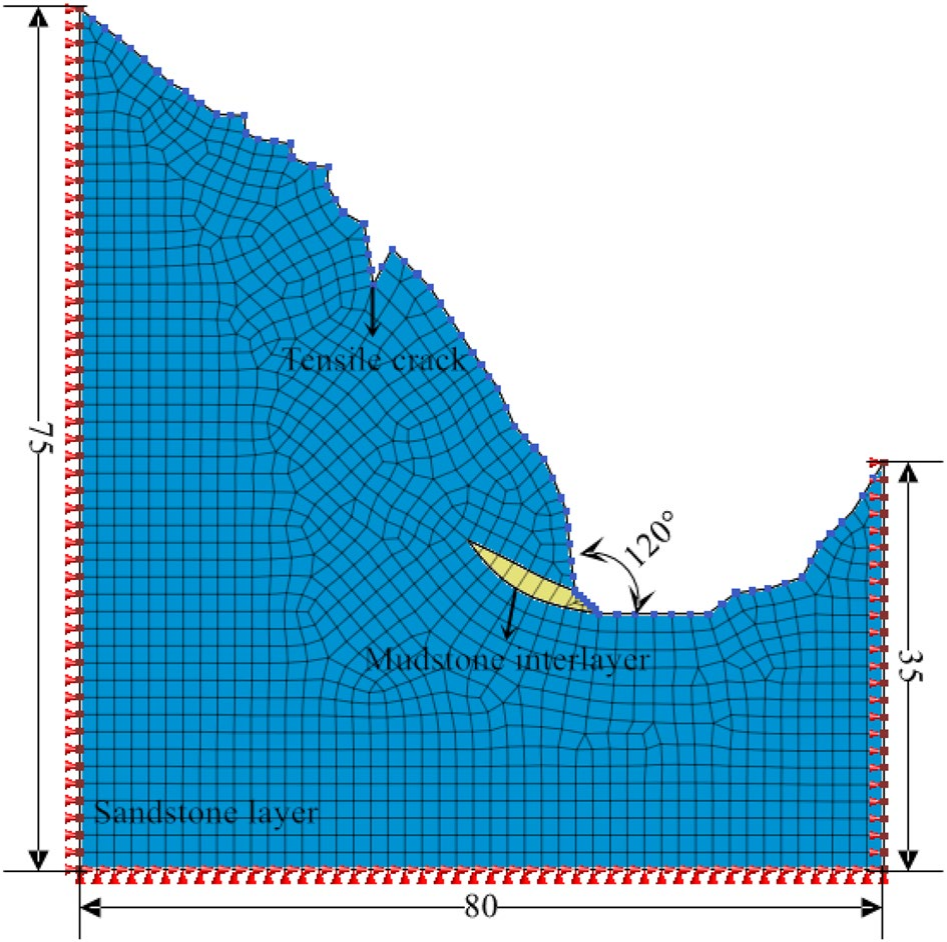
Numerical model of landslide in Huaxi Village, Santun Township.

In order to verify the generalized model, a two-dimensional numerical model was established for the Huaxi Village landslide in Santun Township, Ruyang County (Fig. 14). The total length of the model is 80 m, the total height is 75 m, the slope gradient is 60°, the main lithology is sandstone, there is a mudstone interlayer at the foot of the slope, and there are tensile cracks at the trailing edge. The model is analyzed by Midas GTS NX two-dimensional modeling. In order to simplify the calculation and highlight the regularity, the single-layer bedding rock layer is set for calculation. The model has a total of 2743 nodes and 2829 units; the unit type is a two-dimensional plane strain unit; the soil constitutive model adopts the Mohr–Coulomb model; and the rock strata are set up by direct contact. The rock mass parameters are shown in Table 2, and the working conditions of the fluid–solid coupling simulation analysis are set up (Fig. 18).

**Figure 19:**
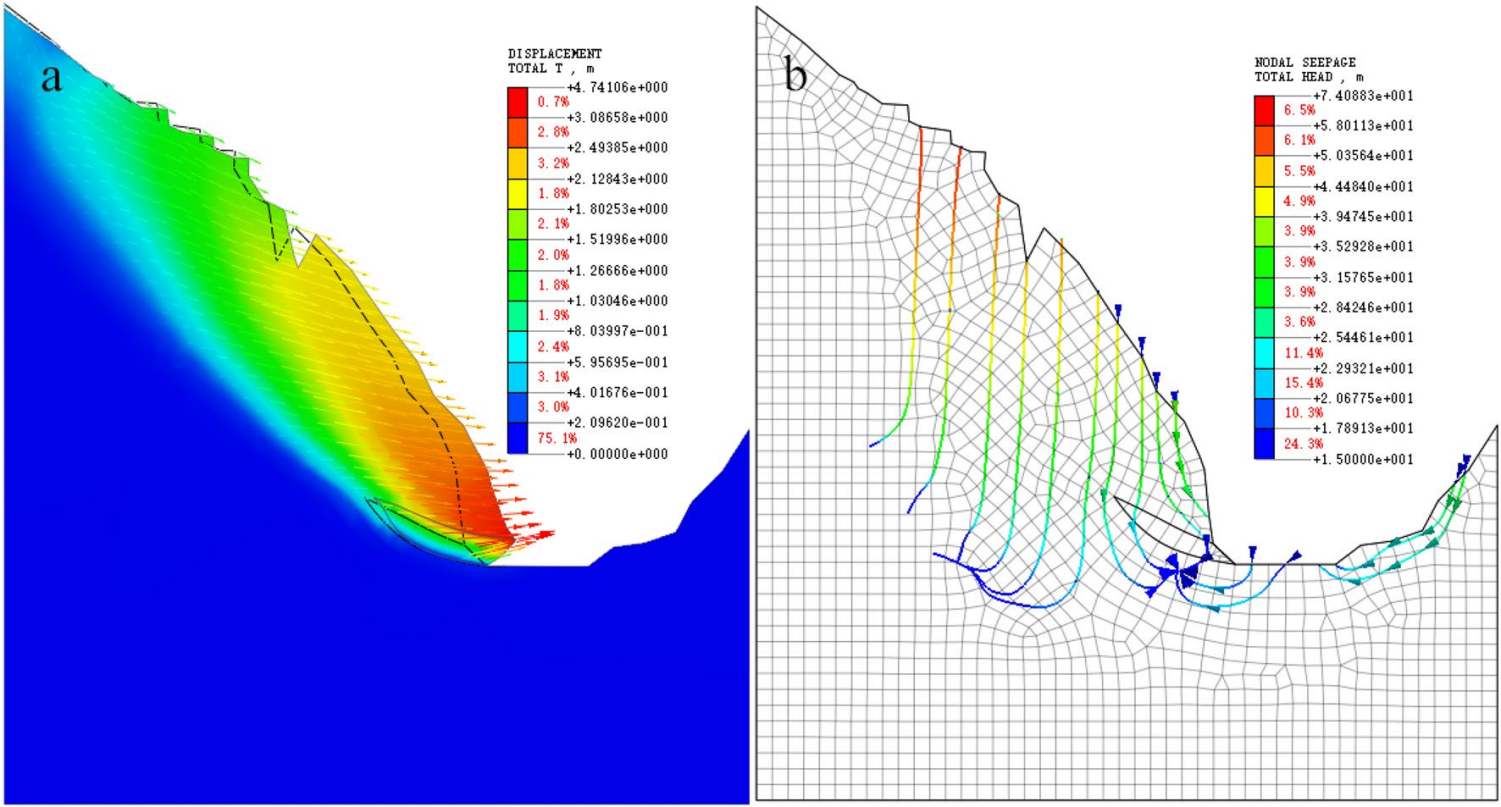
Example slope numerical calculation results: (**a**) displacement cloud map; (**b**) Seepage flow path.

The control group of the example model is the working condition 20 of the generalized model. The model parameter reference Fig. 5 shows that the slope height is 50 m, the slope is 60°, and the inclination angle of the weak interlayer is 20°. The numerical calculation of the of the displacement cloud diagram and seepage flow path of the example slope are shown in Fig. 19. Compared with the generalized model (Fig. 5), the slope type of the Huaxicun landslide is steep, and there is no platform at the top of the slope. The internal deformation of the slope is concentrated between the trailing edge crack and the weak interlayer. With the large-scale slip at the toe of the slope, the trailing edge of the slope is deformed, so the overall deformation area is large. According to the slope surface line and displacement vector direction before and after deformation, the slope slip shear failure along the slope foot is consistent with the failure mode of the generalized model condition 20 (Fig. 8). The stability coefficients before and after rainfall are 1.15 and 0.97, respectively; that is, the natural slope is relatively stable, and the landslide occurs after rainfall. The rainfall infiltration leads to a rapid increase in the groundwater level line inside the slope. The seepage flow path at the weak interlayer is outside the slope, and the drag force on the sliding surface of the slope is outside the slope. Secondly, the infiltration of rainfall increases the water absorption bulk density of weathered sandstone, which makes the friction coefficient between the water-bearing rock soil and the lower non-water-bearing rock soil smaller and the anti-sliding force lower. Due to the traction force formed by the increase in self-weight, the cracks in the upper part of the slope continue to extend, and the slope eventually loses stability.

## Discussion

Based on the disaster-pregnant environment and development characteristics of landslide disasters in the western region of Henan Province, this paper takes the ‘oblique cutting’ type locked rock slope in the layered rock slope as the research object, combines the regional rainfall conditions, and uses the numerical simulation method to study the deformation and failure mechanism and stability influence characteristics of the oblique cutting rock slope in the western region of Henan Province under rainfall conditions. It reveals the instability evolution mechanism of the oblique cutting type locked rock slope under rainfall conditions, and solves the problem that the instability mechanism of the oblique cutting type locked rock slope in the western region of Henan Province is unknown.

It should be pointed out that this paper is a generalized model based on the statistical results of landslide disaster characteristics in the western region of Henan Province. The geometric parameters of the slope, such as the slope height, the slope angle, and the inclination angle of the weak interlayer, are all range values. In order to reduce the computational burden, the representative parameters selected in this paper are divided into larger scales. The purpose is to have a macro-cognition of the failure mode of such slopes and make up for the gaps in related research. In fact, within the given range of slope parameters, increasing the selection density to consider more types of slope parameter combinations can obtain a more detailed and clear instability mechanism of the oblique-cut locking rock slope. In addition, this paper uses the generalized model to carry out theoretical derivation, which has important practical reference value for the identification and disaster prevention of landslides in the study area. However, in terms of theoretical verification, it is limited to comparing with landslide examples, lacking strong support. The follow-up can be further verified by means of physical model construction and a geophysical survey.

## Conclusion

1. In the western region of Henan Province, the oblique-cut locking rock slope is layered, but it does not have the conditions to slide out along the stratum level. The slope body can only slide out through the stratum at a certain angle, and the lithology is mostly medium and high strength rock such as sand mudstone and sandstone. There is a weak interlayer at the toe of the slope. The connection between the weak interlayer and the trailing edge crack is the locking section of the slope. The range is determined by the geometric parameters of the slope and the dip angle of the weak interlayer. The characterization length varies between 9.6 m and 55.7 m, which plays a controlling role in the stability of the slope.
2. Under the condition of rainfall, the oblique-cut locking rock slope in western Henan shows five deformation and failure modes: When the slope is less than 50° and the slope height is less than 50 m, sliding shear failure occurs along the slope foot. When the slope is 50°–55° and the slope height is 50–55 m, sliding shear failure along the plane occurs. Shear failure occurs in the middle of the slope when the slope is 55°–60° and the slope height is 55–60 m. When the dip angle of the weak interlayer is more than 30°, sliding shear failure of the slope foot along the plane occurs. The uplift shear failure of the lower edge of the stratum occurs when the dip angle of the weak interlayer is greater than the internal friction angle of the stratum.
3. The strength reduction method is used to analyze the influence law of the stability of cross-layer oblique locking rock slopes under the influence of different slope geometric factors. It can be seen that when the slope is < 45°, the slope stability coefficient decreases slowly with the increase in slope height. The slope stability coefficient drops sharply when the slope is > 45°. When the dip angle of the weak interlayer is less than 30°, the stability coefficient of the seepage flow path towards the inner slope increases. When the angle of the weak interlayer is more than 30°, the length of the potential locking section increases, and the stability coefficient of the slope increases further. When slope > 55° and slope height > 55 m, the shear stress of the locking section exceeds its shear strength, and the probability of landslide instability is greatly increased.

## Data Availability

The source of the data in this article has been reflected in the text.
